# Trends and Patterns in Oral and Maxillofacial Biopsies in a Tertiary Care Centre of Jharkhand

**DOI:** 10.7759/cureus.115324

**Published:** 2026-08-27

**Authors:** Shipha Akanchha Kujur, Deepali Tirkey, Arvind Kumar, Deepanshu Singh, Sunil Kumar Mahto, Umang K Agrawal

**Affiliations:** 1 Pathology, Rajendra Institute of Medical Sciences, Ranchi, IND; 2 Pathhology, Mahatma Gandhi Memorial Medical College, Jamshedpur, IND; 3 Pathology, Rajendra Institute Of Medical Sciences, Ranchi, IND; 4 Department of General Surgery, Institute of Medical Sciences, Banaras Hindu University, Varanasi, IND; 5 Department of General Surgery, ESIC Medical College and Hospital, Varanasi, IND; 6 Medical School, Nalanda Medical College and Hospital, Patna, IND

**Keywords:** maxillofacial pathology, oral cancers, oral cavity cancer, oral mucosal lesions, oral pyogenic granuloma, oral submucous fibrosis, squamous cell carcinoma (scc)

## Abstract

Introduction

Oral and maxillofacial lesions comprise a diverse spectrum of inflammatory, benign, premalignant, and malignant conditions. Histopathological evaluation is essential for accurate diagnosis and appropriate management.

Aim

This study aimed to evaluate the histopathological spectrum of oral and maxillofacial lesions and describe the distribution of histopathologically diagnosed lesions according to age, sex, anatomical site, and history of tobacco and alcohol consumption.

Methodology

This retrospective descriptive observational study included all oral and maxillofacial biopsy specimens received in the Department of Pathology, Rajendra Institute of Medical Sciences (RIMS), Ranchi, between May 2022 and June 2024. Demographic characteristics, lesion site, histopathological diagnosis, and history of tobacco and alcohol use were retrieved from the pathology records and analyzed.

Results

A total of 141 cases were analyzed (mean age: 31.7 years; range: 22 days-78 years), with a male predominance (70.2%). Neoplastic lesions accounted for 99 cases (70.2%), while 42 (29.8%) were non-neoplastic. Among the 99 neoplastic lesions, 51 (51.5%) were malignant, all diagnosed as squamous cell carcinoma (SCC); 31 (31.3%) were benign, with pyogenic granuloma being the most common; and 17 (17.2%) were premalignant, predominantly oral submucous fibrosis (OSMF). The lower lip (25.5%) and tongue (24.1%) were the most frequently involved sites. Among patients with SCC, 94.1% reported tobacco use and 78.4% reported alcohol consumption, with 62.7% having a history of both exposures.

Conclusion

Neoplastic lesions predominated, with SCC being the most common malignancy and OSMF the most common premalignant lesion. A high proportion of patients with SCC had a history of tobacco and alcohol use. These findings highlight the importance of risk-factor modification and histopathological examination for the early diagnosis and appropriate management of oral and maxillofacial lesions.

## Introduction

Oral cancer is the sixth most common cancer worldwide and represents a major public health burden, particularly in developing countries [1]. The oral cavity and maxillofacial region are continuously exposed to environmental and lifestyle-related carcinogens, making them common sites for a wide spectrum of benign, premalignant, and malignant lesions. In India, oral cancer ranks among the three most common cancers, accounting for more than 30% of all reported malignancies [2]. Tobacco and alcohol consumption remain the principal risk factors, while infections, chronic irritation, and other environmental exposures also contribute to disease development. Histopathological examination remains the gold standard for definitive diagnosis and appropriate patient management [3].

The incidence of oral cancer is approximately 12.6 per 100,000 population [4]. Despite the accessibility of the oral cavity for clinical examination, many lesions continue to be diagnosed at an advanced stage. Regional data on the histopathological spectrum of oral and maxillofacial lesions are limited, particularly from Jharkhand, where a substantial proportion of the population belongs to tribal and socioeconomically disadvantaged communities with high exposure to established risk factors and limited awareness of oral cancer.

The present study aimed to evaluate the histopathological spectrum of oral and maxillofacial lesions diagnosed at a tertiary care center and to describe their distribution according to age, sex, anatomical site, and history of tobacco and alcohol consumption.

## Materials and methods

Study design and setting

This retrospective descriptive observational study was conducted in the Department of Pathology at Rajendra Institute of Medical Sciences (RIMS), Ranchi, Jharkhand, India. Histopathological records of oral and maxillofacial biopsy specimens received between May 2022 and June 2024 were reviewed.

Study population

Pathology records of oral and maxillofacial biopsy specimens received during the study period were retrospectively reviewed. Cases were considered eligible if they represented primary, untreated, histopathologically confirmed benign, premalignant, or malignant oral and maxillofacial lesions. Demographic details, anatomical site, histopathological diagnosis, and history of tobacco and/or alcohol consumption were retrieved from the available pathology records using a structured data collection proforma. Repeat biopsy specimens, previously diagnosed recurrent lesions, and cases with incomplete clinical or histopathological records were excluded. Following application of these predefined eligibility and exclusion criteria, 141 cases were included in the final analysis.

Data collection

Data were extracted retrospectively using a structured data collection proforma. The variables recorded included age, sex, anatomical site of the lesion, histopathological diagnosis, and history of tobacco and/or alcohol consumption. Histopathological diagnoses were categorized as benign, premalignant, or malignant according to established diagnostic criteria.

Statistical analysis

Data were entered and summarized using Microsoft Excel (Microsoft Corporation, Redmond, WA, USA). Categorical variables were summarized using frequencies and percentages. The distribution of oral and maxillofacial lesions was described according to demographic characteristics, anatomical site, histopathological diagnosis, and relevant exposure history.

## Results

**Figure 1:**
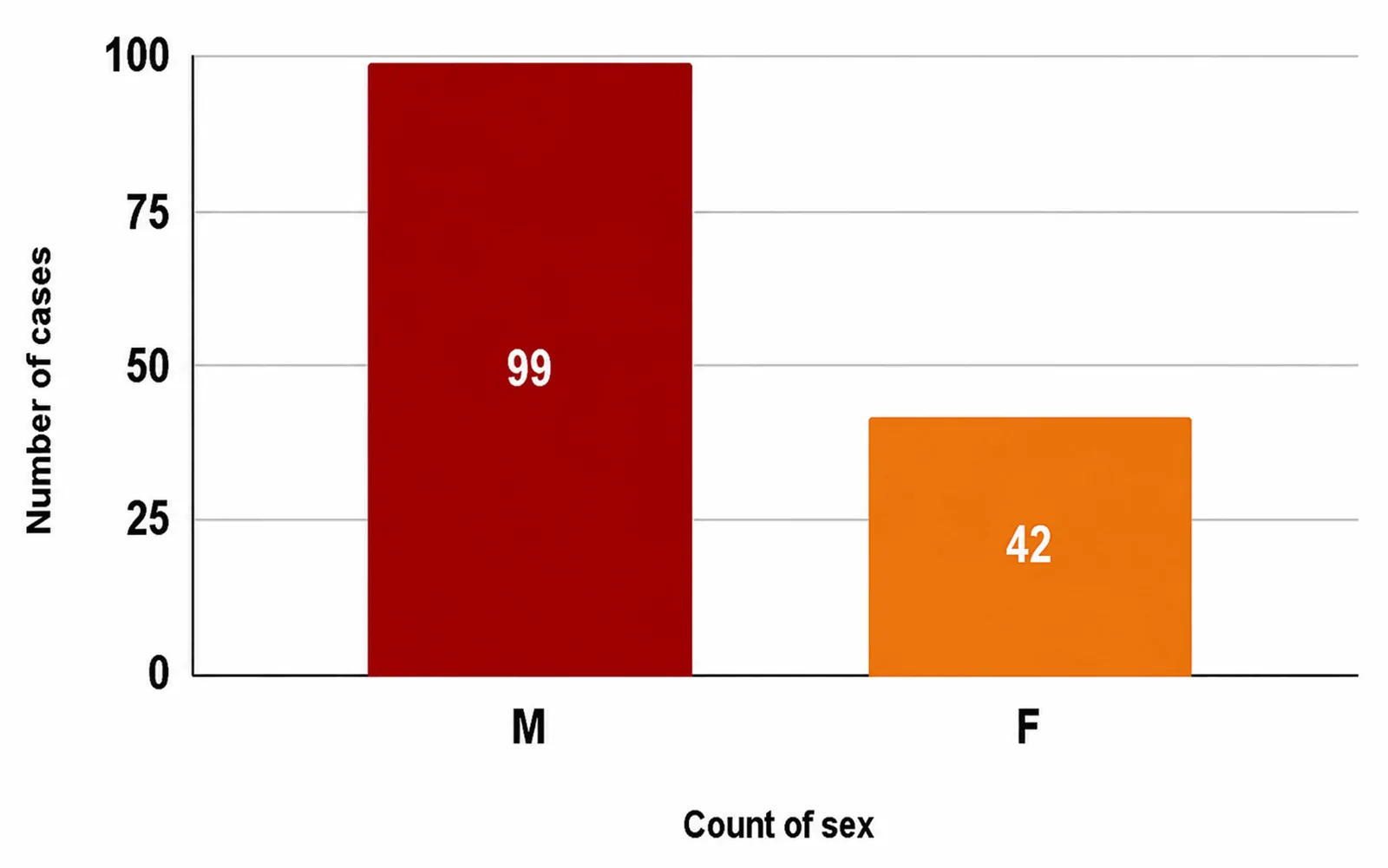
Sex distribution of the study participants.

A total of 141 participants were included, with a mean age of 31.7 years (range: 22 days to 78 years). The study population comprised 99 males (70.2%) and 42 females (29.8%) (Figure 1).

The highest frequency was observed in the 41-50-year age group (27 cases, 19.1%), while the lowest was among infants aged <1 year (one case, 0.7%) (Table 1). 

**Table 1:** Age distribution of the study participants

Age group (years)	Frequency (n)	Percentage (%)
<1 year	1	0.7
1–10	10	7.1
11–20	17	12.1
21–30	22	15.6
31–40	20	14.2
41–50	27	19.1
51–60	20	14.2
61–70	9	6.4
71–80	15	10.6
Total	141	100.0

The lower lip was the most commonly affected site (36 cases, 25.5%), followed by the tongue (34 cases, 24.1%) (Table 2).

**Table 2:** Distribution of oral and maxillofacial lesions according to the anatomical site

Site	Frequency (n)	Percentage (%)
Lower lip	36	25.5
Tongue	34	24.1
Left buccal mucosa	28	19.9
Right buccal mucosa	17	12.1
Upper lip	9	6.4
Mandible	8	5.7
Jaw	3	2.1
Right upper alveolus	2	1.4
Maxilla	2	1.4
Right lower alveolus	1	0.7
Left angle of mouth	1	0.7
Total	141	100.0

**Figure 2:**
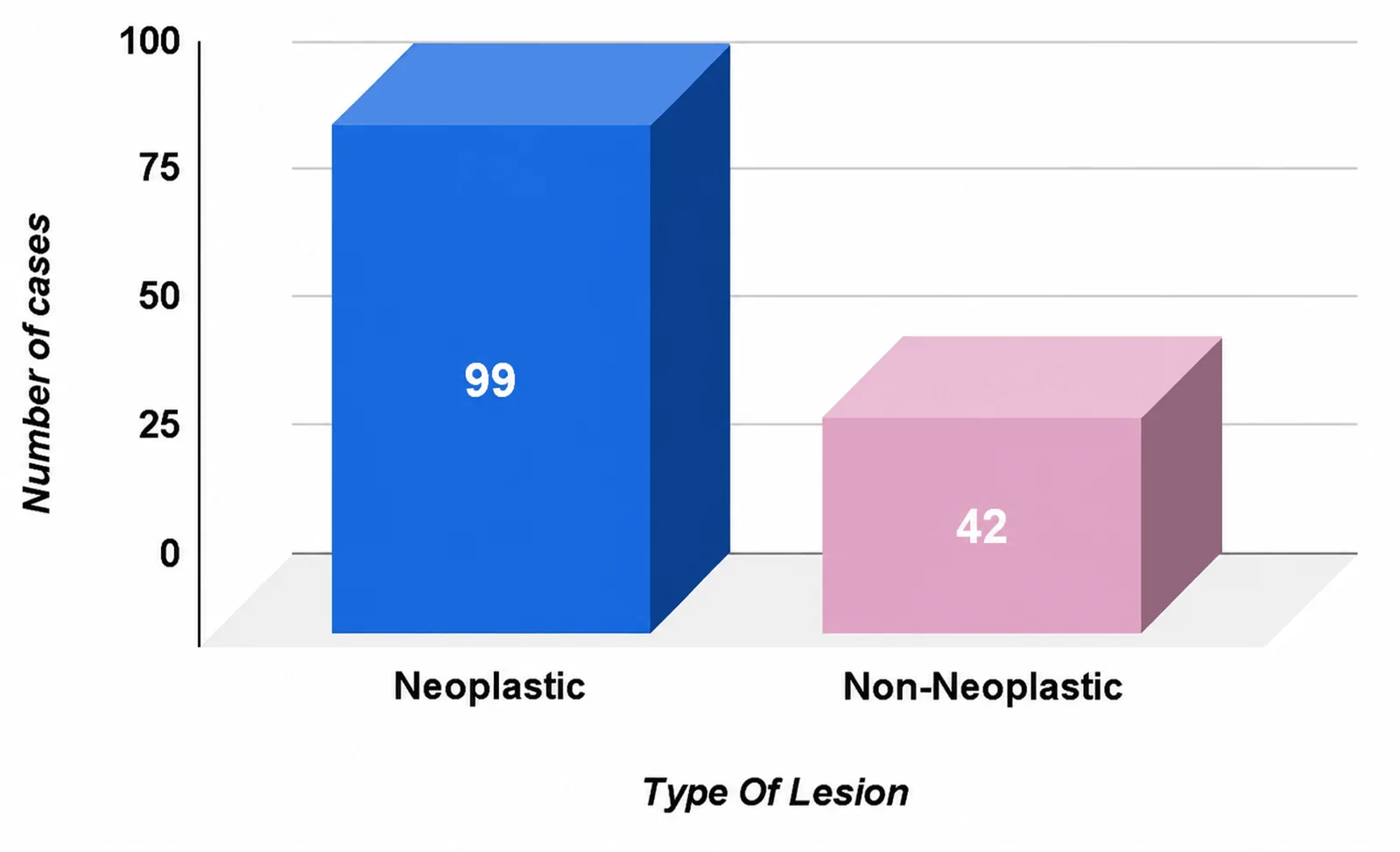
Distribution of neoplastic and non-neoplastic lesions.

Overall, neoplastic lesions accounted for 99 cases (70.2%), while non-neoplastic lesions comprised 42 cases (29.8%) (Figure 2). Both neoplastic and non-neoplastic lesions were more frequent in males.

Among the 42 non-neoplastic lesions, chronic inflammatory lesions were the most common, accounting for 21 cases (50.0%), followed by retention cysts (10 cases, 23.8%) and sialadenosis (nine cases, 21.4%). Dentigerous cyst and vascular malformation were each identified in one case (2.4%) (Table 3).

**Table 3:** Distribution of non-neoplastic lesions based on histopathological diagnosis

Histopathological diagnosis	n	%
Chronic inflammatory lesions*	21	50.0
Retention cyst	10	23.8
Sialadenosis	9	21.4
Dentigerous cyst	1	2.4
Vascular malformation	1	2.4
Total	42	100.0

**Figure 3:**
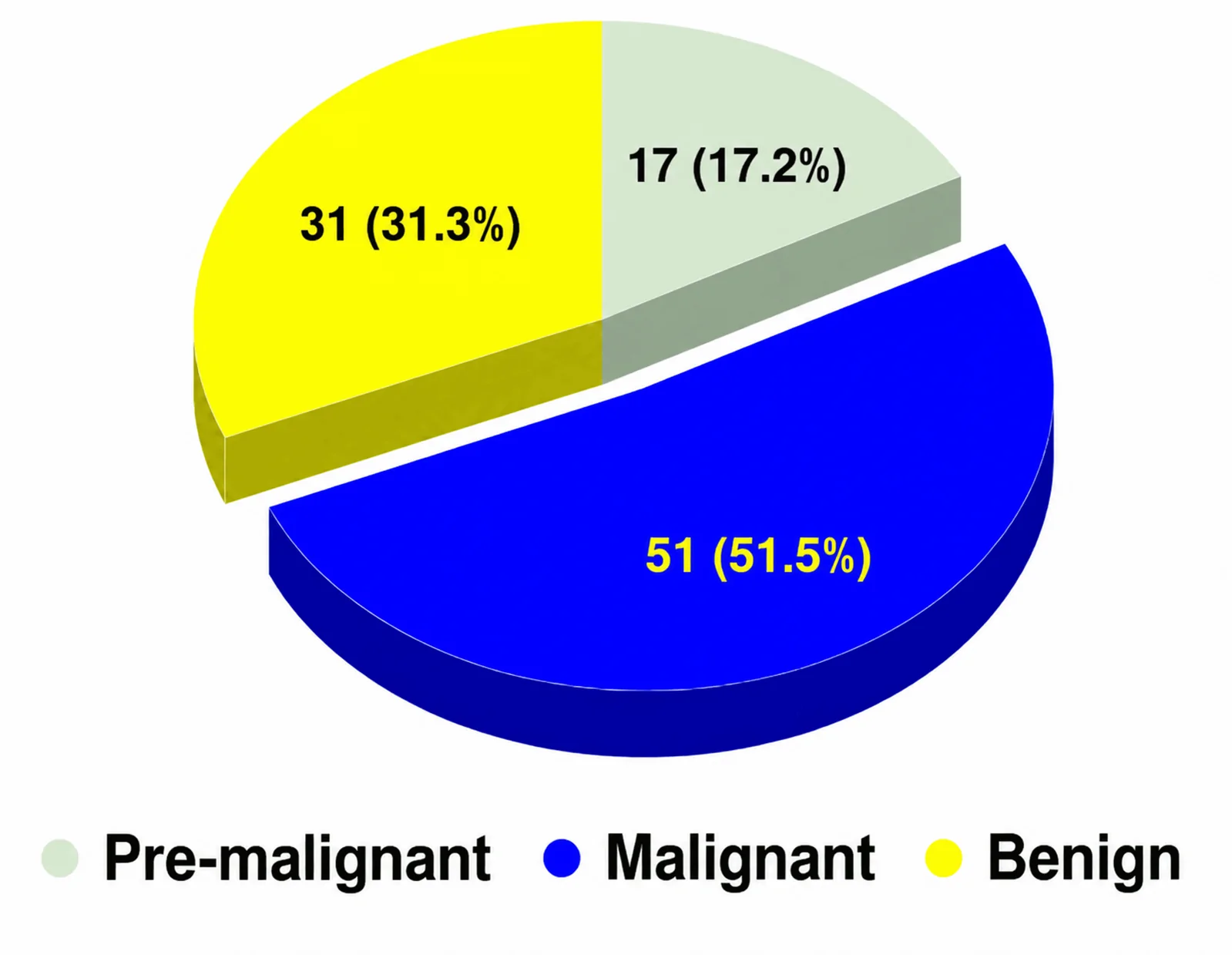
Pie chart showing different categories of neoplastic lesions.

Among the 99 neoplastic lesions, 51 (51.5%) were malignant, 31 (31.3%) were benign, and 17 (17.2%) were premalignant (Figure 3).

Malignant and premalignant lesions were most frequently observed in individuals aged 51-70 years, whereas benign lesions and non-neoplastic lesions were more common in the 31-40-year age group. Benign and premalignant lesions showed a relatively balanced sex distribution, while malignant lesions demonstrated a marked male predominance.

Among the benign lesions, pyogenic granuloma was the most common histopathological diagnosis (7/31, 22.6%), characterized by lobular capillary proliferation, followed by fibroepithelial polyp (6/31, 19.4%), ameloblastoma and squamous papilloma (5/31 each, 16.1%), hemangioma (3/31, 9.7%), mucocele and benign cystic lesion (2/31 each, 6.5%), and pleomorphic adenoma (1/31, 3.2%) (Table 4).

**Table 4:** Distribution of benign neoplastic lesions according to histopathological diagnosis (N = 31)

Histopathological diagnosis	n	%
Pyogenic granuloma	7	22.6
Fibroepithelial polyp	6	19.4
Ameloblastoma	5	16.1
Squamous papilloma	5	16.1
Hemangioma	3	9.7
Mucocele	2	6.5
Benign cystic lesion	2	6.5
Pleomorphic adenoma	1	3.2
Total	31	100.0

Among the premalignant lesions, oral submucous fibrosis (OSMF) was the most common histopathological diagnosis (7/17, 41.2%), followed by moderate dysplasia (5/17, 29.4%), mild dysplasia (4/17, 23.5%), and leukoplakia (1/17, 5.9%) (Table 5).

**Table 5:** Distribution of premalignant neoplastic lesions according to histopathological diagnosis (N = 17)

Histopathological diagnosis	n	%
Oral submucous fibrosis	7	41.2
Moderate dysplasia	5	29.4
Mild dysplasia	4	23.5
Leukoplakia	1	5.9
Total	17	100.0

**Figure 4:**
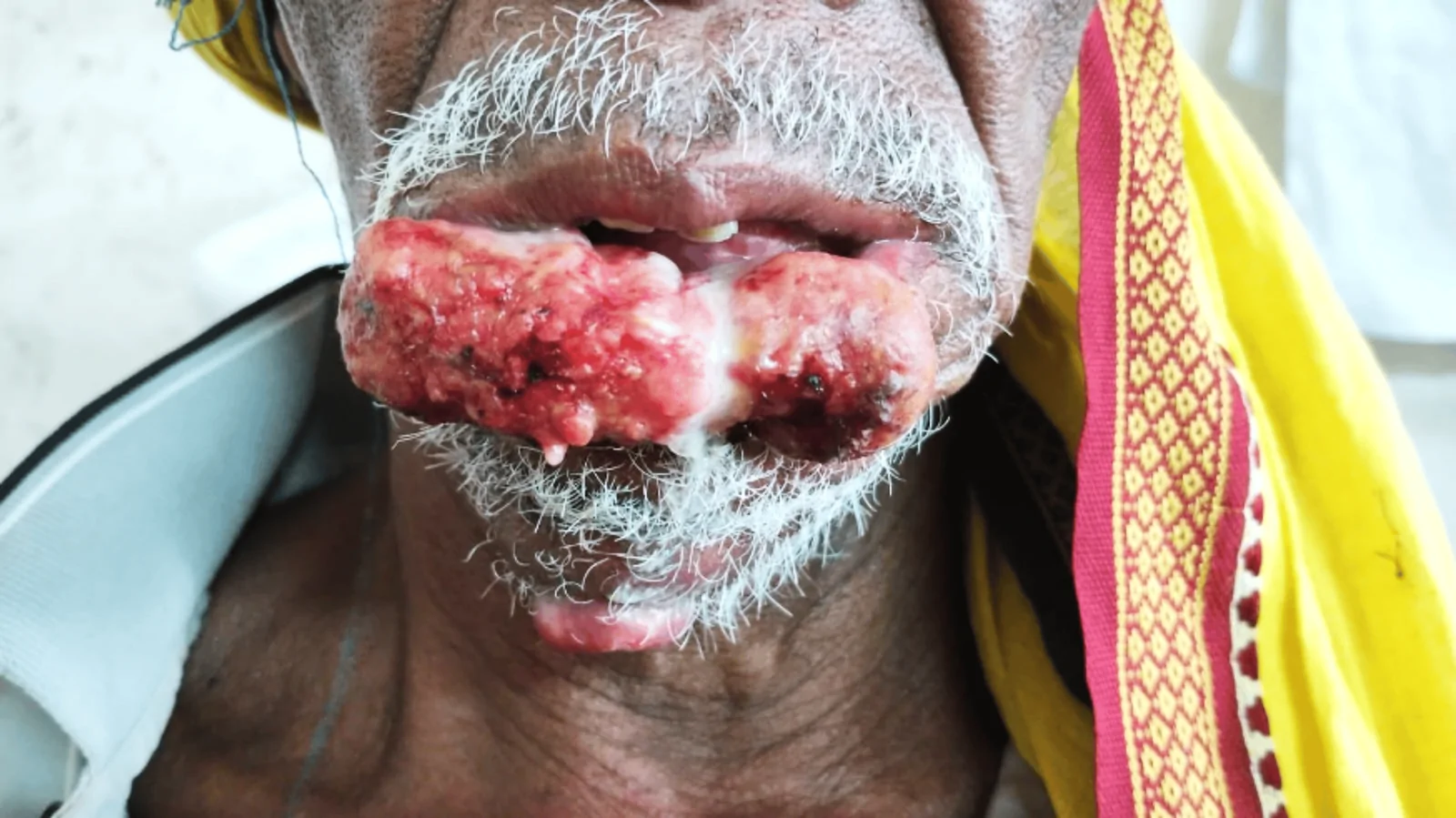
Gross clinical photograph showing a large exophytic, ulceroproliferative lesion involving the lower lip. Histopathological examination confirmed well-differentiated squamous cell carcinoma.

**Figure 5:**
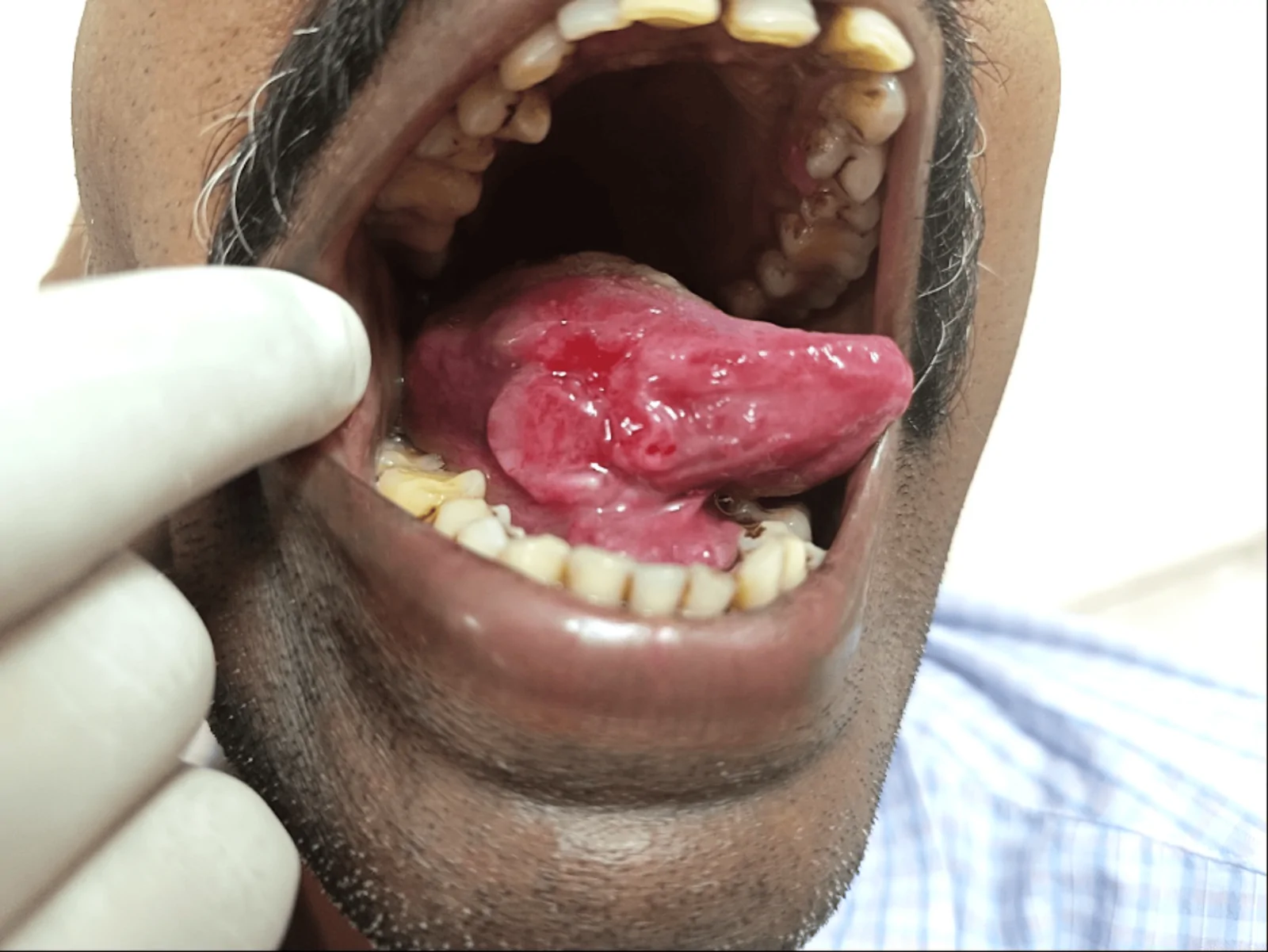
Gross clinical photograph showing an irregular ulceroproliferative lesion along the left lateral border of the tongue. Histopathological examination confirmed well-differentiated squamous cell carcinoma.

**Figure 6:**
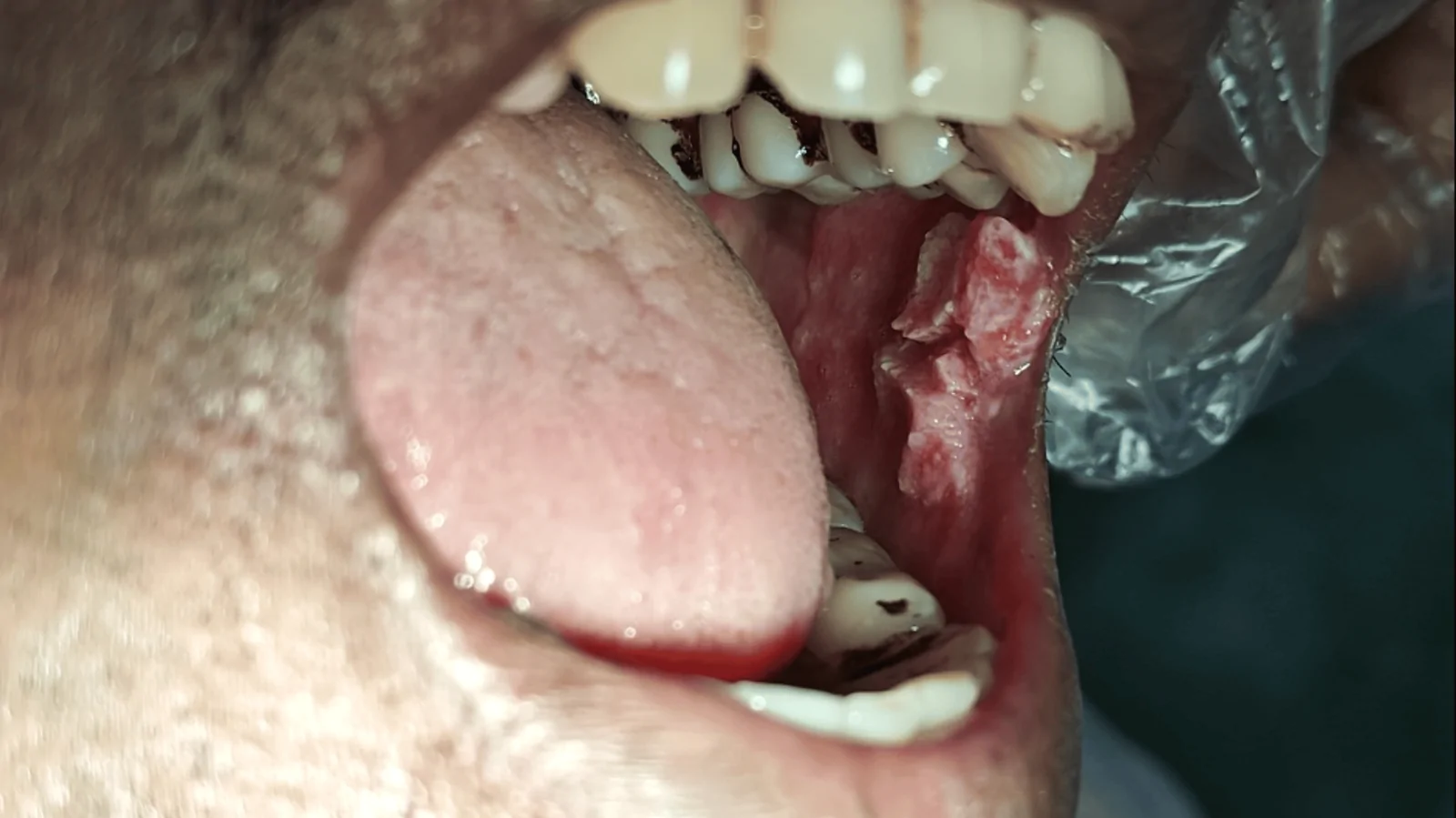
Gross clinical photograph showing an ulceroproliferative lesion involving the left angle of the mouth extending to the adjacent buccal mucosa. Histopathological examination confirmed well-differentiated squamous cell carcinoma.

**Figure 7:**
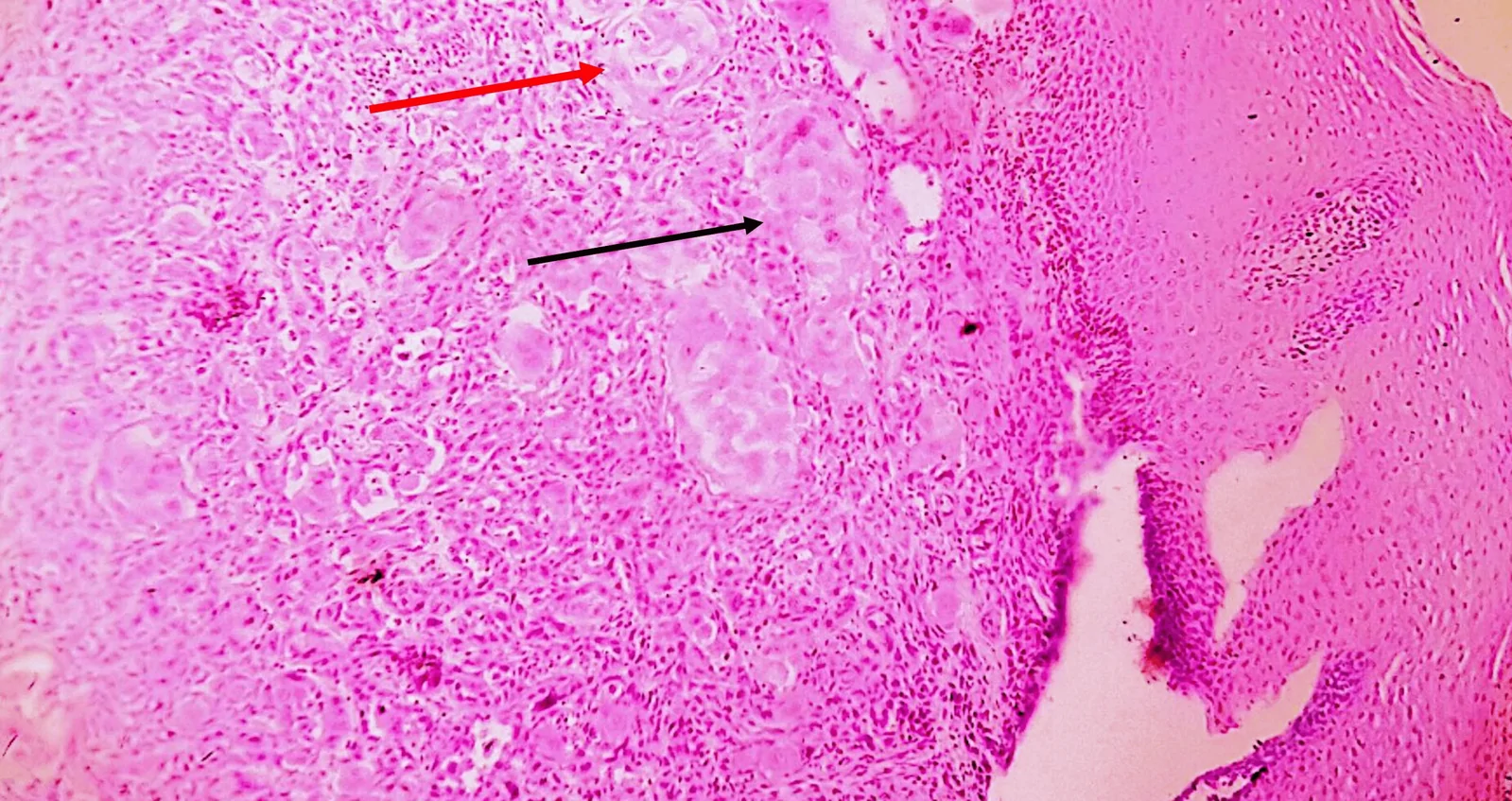
Photomicrograph of well-differentiated squamous cell carcinoma showing tumor nests (black arrow) and keratin pearls (red arrow) (H&E, ×100).

**Figure 8:**
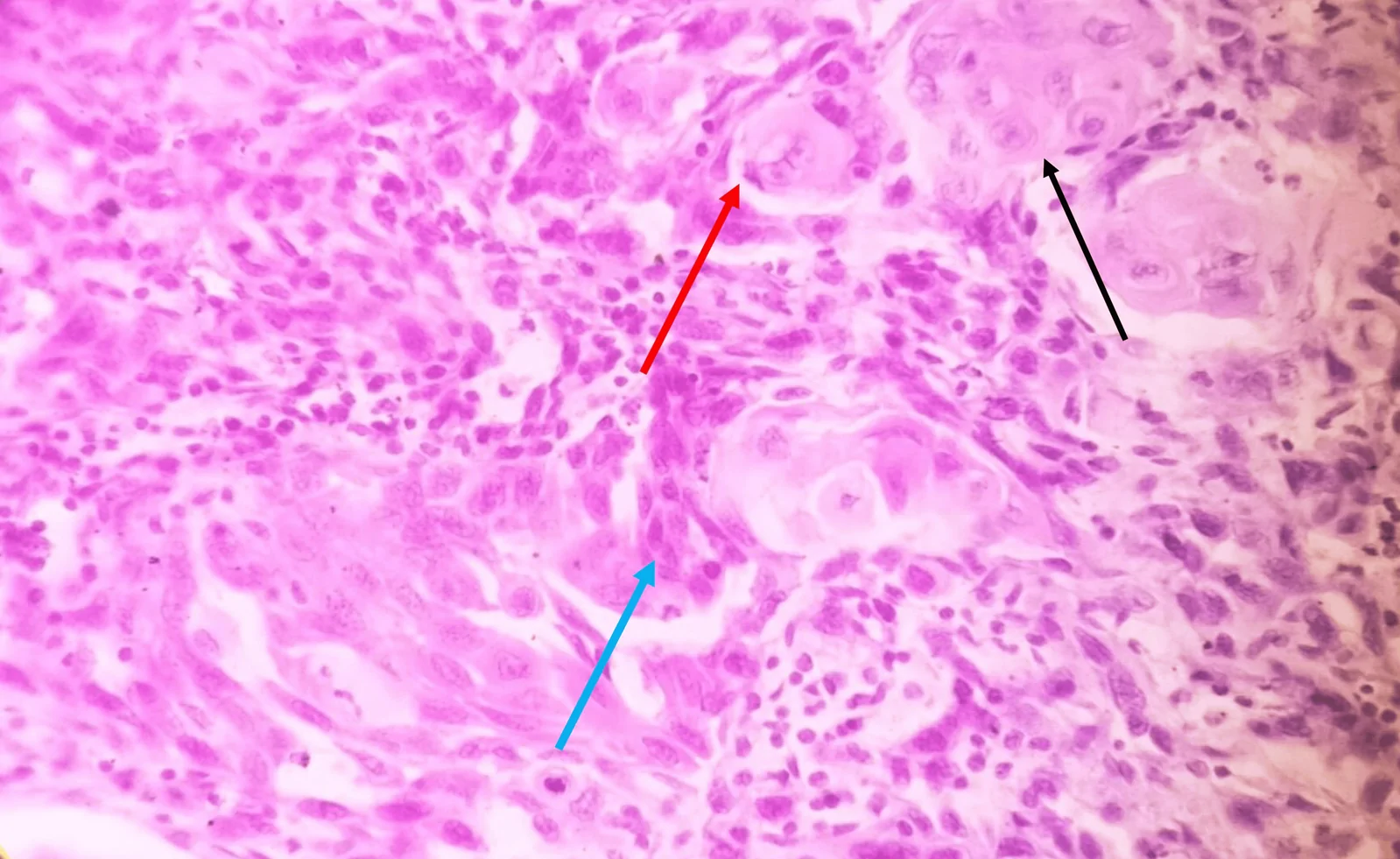
Photomicrograph of well-differentiated squamous cell carcinoma showing malignant squamous cells (black arrow), keratin pearl (red arrow), and nuclear pleomorphism (blue arrow) (H&E, ×400).

All malignant lesions were diagnosed as SCC (51 cases, 36.2% of all lesions). Representative gross photographs are shown in Figures 4-6, demonstrating the characteristic ulcerative and exophytic morphology. Histopathological examination revealed nests and lobules of atypical squamous cells with keratin pearl formation (Figures 7, 8). 

Among patients with SCC, 48 (94.1%) reported tobacco use, including 40 smokers (83.3%) and eight users of smokeless tobacco (gutkha/paan, 16.7%). Alcohol use was reported in 40 patients, of whom 32 also used tobacco, indicating frequent co-exposure to these established risk factors.

## Discussion

Malignant lesions were more common in older individuals (mean age 49.5 years), with SCC showing a mean age of 56.65 years, consistent with previous reports on oral cancer [5]. Histopathological examination remains the diagnostic gold standard when interpreted alongside clinical findings and ancillary investigations. Assessment of the distribution and patterns of oral lesions in hospital-based studies can provide useful information for local healthcare planning [6]. In our cohort, a proportion of patients presented with longstanding oral lesions and features suggestive of advanced local disease.

Ameloblastoma predominantly involved the mandible (80%), with the remaining cases arising in the maxilla, consistent with the reported distribution of approximately 80% in the mandible and 20% in the maxilla [7].

Smokeless tobacco use is strongly associated with OSMF and leukoplakia, whereas smoking is more closely associated with oral SCC [8]. Similarly, most patients with OSMF and leukoplakia in our study had a history of chronic smokeless tobacco use, while the majority of patients with SCC reported long-term tobacco consumption, often for 30-40 years.

The tongue, lower lip, and floor of the mouth are recognized as the most common sites for oral SCC [9]. In our study, the tongue was the most frequent site, followed by the lower lip, buccal mucosa, and mandible. Most patients with premalignant and malignant lesions also reported paan chewing, consistent with the established role of tobacco and areca nut as risk factors for oral carcinogenesis [10]. Although SCC predominantly affected males aged ≥50 years, younger patients were also identified, with the youngest affected individual being 30 years old, consistent with previous reports describing SCC among younger adults [11]. We also observed frequent use of smokeless tobacco (khaini) among tribal women, commonly attributed to relief of dental pain.

Patients commonly presented with features suggestive of oral malignancy, including persistent ulcers, oral patches, swellings, pain, restricted mouth opening, dysphagia, and bleeding, in agreement with previously described warning signs [12].

The male-to-female ratio in our study was 2.3:1, comparable to that reported by Malhotra et al. but lower than that observed in some other populations [13]. Tobacco use was also more common among males (3.4:1), likely reflecting greater exposure to established risk factors.

Most patients with malignant lesions belonged to lower socioeconomic and tribal communities of Jharkhand and presented with advanced disease, suggesting possible disparities in awareness, healthcare access, and early diagnosis [2].

Combined exposure to tobacco and alcohol was common among patients with SCC, consistent with the well-established synergistic effect of these risk factors in oral carcinogenesis [14].

Previous studies have reported varying willingness among women to quit tobacco use across different Indian states [15]. In contrast, female tobacco users with benign and non-neoplastic lesions in our cohort generally showed reluctance to quit, whereas patients with malignant lesions were more willing to discontinue tobacco use following diagnosis.

Limitations

This retrospective, descriptive, single-centre, hospital-based study may limit the generalizability of the findings. Selection bias is possible as only biopsied lesions were included; therefore, the observed frequencies should not be interpreted as population-level prevalence or incidence. The absence of a comparison group limits assessment of associations between tobacco, alcohol, paan chewing, and specific lesions. Archived records may have incomplete information on variables, such as lesion duration, clinical appearance, lesion size, and risk factors, and tobacco and alcohol histories, may have been incompletely or inconsistently documented. The absence of long-term follow-up precluded assessment of clinical outcomes. Larger multicentre prospective studies with standardized data collection and appropriate comparison groups are needed to validate these findings.

## Conclusions

Oral and maxillofacial lesions comprise a wide spectrum of inflammatory, benign, premalignant, and malignant conditions. Histopathological examination of oral biopsies remains indispensable for accurate diagnosis and appropriate management. In this hospital-based series, malignant lesions showed a male predominance, with the tongue being the most frequently involved site and SCC representing the most common malignancy. Among benign neoplastic lesions, pyogenic granuloma, squamous papilloma, and fibroepithelial polyp were the most frequent diagnoses, whereas non-neoplastic lesions commonly presented as swellings and malignant lesions as ulceroproliferative growths. A high proportion of patients with premalignant and malignant lesions had a documented history of tobacco use, underscoring the importance of tobacco-control measures and public awareness. These findings describe the pattern of lesions among biopsied cases encountered at a tertiary care centre and should not be interpreted as population-level prevalence or incidence estimates. Careful clinicopathological correlation and early evaluation of persistent oral lesions remain essential for accurate diagnosis and appropriate patient management.
